# Author Correction: Nutrients cause grassland biomass to outpace herbivory

**DOI:** 10.1038/s41467-021-20985-z

**Published:** 2021-01-20

**Authors:** E. T. Borer, W. S. Harpole, P. B. Adler, C. A. Arnillas, M. N. Bugalho, M. W. Cadotte, M. C. Caldeira, S. Campana, C. R. Dickman, T. L. Dickson, I. Donohue, A. Eskelinen, J. L. Firn, P. Graff, D. S. Gruner, R. W. Heckman, A. M. Koltz, K. J. Komatsu, L. S. Lannes, A. S. MacDougall, J. P. Martina, J. L. Moore, B. Mortensen, R. Ochoa-Hueso, H. Olde Venterink, S. A. Power, J. N. Price, A. C. Risch, M. Sankaran, M. Schütz, J. Sitters, C. J. Stevens, R. Virtanen, P. A. Wilfahrt, E. W. Seabloom

**Affiliations:** 1 Department of Ecology, Evolution and Behavior, University of Minnesota, St. Paul, MN USA; 2 Helmholtz Center for Environmental Research – UFZ, Department of Physiological Diversity, Permoserstrasse 15, 04318 Leipzig, Germany; 3 German Centre for Integrative Biodiversity Research (iDiv), Deutscher Platz 5e, 04103 Leipzig, Germany; 4 Martin Luther University Halle-Wittenberg, am Kirchtor 1, 06108 Halle (Saale), Germany; 5 Department of Wildland Resources and the Ecology Center, Utah State University, Logan, UT USA; 6 Department of Physical and Environmental Sciences, University of Toronto - Scarborough, Toronto, ON Canada; 7 Centre for Applied Ecology (CEABN-InBIO), School of Agriculture, University of Lisbon, Tapada da Ajuda, Lisbon, Portugal; 8 Department of Biological Sciences, University of Toronto - Scarborough, Toronto, ON Canada; 9 Forest Research Centre, School of Agriculture, University of Lisbon, Tapada da Ajuda, Lisbon, Portugal; 10 IFEVA, Universidad de Buenos Aires, CONICET, Facultad de Agronomía, Buenos Aires, Argentina; 11 School of Life and Environmental Sciences, The University of Sydney, Sydney, NSW Australia; 12 Department of Biology, University of Nebraska at Omaha, Omaha, NE USA; 13 Department of Zoology, School of Natural Sciences, Trinity College Dublin, Dublin, Ireland; 14 Department of Ecology & Genetics, University of Oulu, Oulu, Finland; 15 School of Earth, Environmental and Biological Sciences, Queensland University of Technology, Brisbane, QLD Australia; 16 Department of Entomology, University of Maryland, College Park, MD USA; 17 Department of Biology, University of North Carolina, Chapel Hill, NC USA; 18 Department of Integrative Biology, University of Texas, Austin, TX USA; 19 Department of Biology, Washington University in St. Louis, St. Louis, MO USA; 20 Smithsonian Environmental Research Center, Edgewater, MD USA; 21 Department of Biology and Animal Sciences, São Paulo State University - UNESP, São Paulo, Brazil; 22 Department of Integrative Biology, University of Guelph, Guelph, ON Canada; 23 Department of Biology, Texas State University, San Marcos, TX USA; 24 School of Biological Sciences, Monash University, Clayton Campus, Clayton, VIC Australia; 25 Department of Biology, Benedictine College, Atchison, KS USA; 26 Department of Biology, IVAGRO, University of Cádiz, Cádiz, Spain; 27 Department of Biology, Vrije Universiteit Brussel, Brussels, Belgium; 28 Hawkesbury Institute for the Environment, Western Sydney University, Richmond, NSW Australia; 29 Institute of Land, Water and Society, Charles Sturt University, Albury, NSW Australia; 30 Swiss Federal Institute for Forest, Snow and Landscape Research, Birmensdorf, Switzerland; 31 National Centre for Biological Sciences, TIFR, Bengaluru, India; 32 School of Biology, University of Leeds, Leeds, UK; 33 Lancaster Environment Centre, Lancaster University, Lancaster, UK; 34 Department of Disturbance Ecology, University of Bayreuth, Bayreuth, Germany

**Keywords:** Community ecology, Grassland ecology

Correction to: *Nature Communications* 10.1038/s41467-020-19870-y, published online 27 November 2020.

The original version of this Article contained an error in the author affiliations. The affiliation of Martin Schütz with Swiss Federal Institute for Forest, Snow and Landscape Research WSL, Zuercherstrasse 111, 8903 Birmensdorf, Switzerland was inadvertently omitted. Martin Schütz was incorrectly associated with Lancaster Environment Centre, Lancaster University, Lancaster, UK. This has now been corrected in both the PDF and HTML versions of the Article.

