# Supplementary material for: Nutrients cause grassland biomass to outpace herbivory
Source: Nat Commun. 2020 Nov 27;11:6036. doi: 10.1038/s41467-020-19870-y (PMC7695826; doi:10.1038/s41467-020-19870-y)
Supplement: Supplementary file 1 — Supplementary Information [file 41467_2020_19870_MOESM1_ESM.pdf]

Supplementary Table 1. Nutrient Network sites included in the current study. Data shown are location, duration of experimental treatment, whether domestic herbivores were present (all sites had wild herbivores), the herbivore index value, and the modeled potential grazer biomass value.

Site number	Site code	Continent	Country	Latitude	Longitude	Exp. years	Domestic herbivores	Herbivore index (square root)	Modeled grazer biomass
1	sereng.tz	Africa	Tanzania	-2.255	34.513	4	No	5.39	1.69
2	gilb.za	Africa	Zambia	-29.284	30.292	3	No	2.24	3.39
3	azi.cn	Asia	China	33.670	101.870	4	Yes	2.00	1.73
4	kibber.in	Asia	India	32.320	78.010	6	Yes	4.69	0.01
5	bogong.au	Australia	Australia	-36.874	147.254	10	No	2.24	2.57
6	burrawan.au	Australia	Australia	-27.735	151.140	11	Yes	3.61	1.69
7	kiny.au	Australia	Australia	-36.200	143.750	11	No	2.24	1.51
8	mtca.au	Australia	Australia	-31.782	117.611	10	Yes	3.00	1.09
9	ping.au	Australia	Australia	-32.496	116.973	5	No	4.00	1.75
10	pinj.au	Australia	Australia	-27.531	152.923	5	Yes	2.45	1.09
11	yarra.au	Australia	Australia	-33.610	150.730	5	No	2.45	2.02
12	frue.ch	Europe	Switzerland	47.113	8.542	7	No	1.73	2.00
13	valm.ch	Europe	Switzerland	46.631	10.372	10	No	3.16	2.16
14	bayr.de	Europe	Germany	49.917	11.583	3	No	NA	1.24
15	pape.de	Europe	Germany	53.086	7.473	6	No	2.65	0.34
16	kilp.fi	Europe	Finland	69.057	20.875	5	No	3.16	0.33
17	saana.fi	Europe	Finland	69.037	20.843	4	No	NA	0.33
18	cereep.fr	Europe	France	48.280	2.660	6	No	NA	0.47
19	burren.ie	Europe	Ireland	53.072	-8.993	3	No	NA	0.40
20	comp.pt	Europe	Portugal	38.829	-8.791	7	No	1.00	0.78
21	hero.uk	Europe	United Kingdom	51.411	-0.639	5	No	2.83	0.34
22	lancaster.uk	Europe	United Kingdom	53.986	-2.628	9	Yes	3.00	0.41
23	rook.uk	Europe	United Kingdom	51.406	-0.644	5	No	2.83	0.34
24	cowi.ca	N America	Canada	48.809	-123.630	11	No	1.73	2.01
25	koffler.ca	N America	Canada	44.024	-79.536	8	No	2.83	0.35
26	arch.us	N America	United States	27.150	-81.183	3	No	2.83	1.22
27	barta.us	N America	United States	42.245	-99.652	4	No	3.00	2.12
28	bldr.us	N America	United States	39.972	-105.234	8	No	1.41	1.96
29	bnch.us	N America	United States	44.277	-121.968	11	No	2.83	3.66
30	cbgb.us	N America	United States	41.785	-93.385	9	No	3.16	1.84
31	cdcr.us	N America	United States	45.425	-93.212	11	No	2.83	0.37
32	cdpt.us	N America	United States	41.200	-101.630	11	No	2.65	3.09
33	elliot.us	N America	United States	32.875	-117.052	11	No	2.83	0.58

34	glcr.us	N America	United States	41.339	-96.140	2	No	2.65	2.84
35	hall.us	N America	United States	36.872	-86.702	7	No	2.24	0.39
36	hart.us	N America	United States	42.724	-119.498	5	No	3.32	0.23
37	hnvr.us	N America	United States	43.419	-72.138	3	No	2.83	0.41
38	hopl.us	N America	United States	39.013	-123.060	11	No	2.45	1.64
39	kbs.us	N America	United States	42.409	-85.391	4	No	NA	1.08
40	konz.us	N America	United States	39.071	-96.583	11	No	1.41	3.73
41	look.us	N America	United States	44.205	-122.128	7	No	2.83	0.42
42	mcla.us	N America	United States	38.864	-122.406	11	No	2.24	1.64
43	sage.us	N America	United States	39.430	-120.240	6	No	3.00	1.89
44	saline.us	N America	United States	39.050	-99.100	8	No	1.41	3.96
45	sava.us	N America	United States	33.344	-81.651	5	No	2.24	0.43
46	sgs.us	N America	United States	40.817	-104.767	11	No	3.74	1.92
47	shps.us	N America	United States	44.243	-112.198	9	Yes	2.65	1.23
48	sier.us	N America	United States	39.236	-121.284	11	No	2.00	1.89
49	smith.us	N America	United States	48.207	-122.625	8	No	2.65	2.01
50	spin.us	N America	United States	38.136	-84.501	11	No	2.00	0.48
51	trel.us	N America	United States	40.075	-88.829	9	No	2.24	0.63
52	tyso.us	N America	United States	38.519	-90.565	4	No	2.00	0.80
53	ufrec.us	N America	United States	27.433	-81.917	2	No	NA	1.22
54	unc.us	N America	United States	36.008	-79.020	4	No	2.00	0.55
55	chilcas.ar	S America	Argentina	-36.276	-58.266	6	No	0.00	0.74
56	marc.ar	S America	Argentina	-37.715	-57.425	7	No	2.83	0.59
57	potrok.ar	S America	Argentina	-51.916	-70.407	3	Yes	2.83	2.17
58	lagoas.br	S America	Brazil	-20.983	-51.799	3	No	NA	1.38

Supplementary Table 2. Description of exceptions to the fence design; sites not included in this list have standard design.

Site code	Fence exception description	Herbivores excluded
bayr.de	1.5 m high fences with 12.7 x 12.7 mm wire mesh and wooden posts. Entire site is fenced to exclude deer; other wild vertebrate herbivores are still present on site.	Wild herbivore
cdpt.us	1.5 m of 10 cm mesh cattle panels, with hardware cloth up to 50 cm from ground level.	Wild herbivores
hall.us	Similar to NutNet standard with two modifications: 3.6 cm hardware cloth and 1.5 m high fences.	Wild herbivores
hart.us	Similar to NutNet standard but instead of 4 wires strung above the hardware cloth, we covered the entire top half of the fence with hardware cloth.	Wild herbivores
lancaster.uk	Similar to NutNet standard but top strand at 1.2 m.	Sheep, wild herbivores
sereng.tz	Posts are made of large steel pipes buried to a depth of approximately 1 m and reinforced with concrete. Fences are approximately 1.3 m tall and made of thick wire gauge fence material that has been threaded through a piece of rebar that has been welded to the steel metal posts.	Wild herbivores
shps.us	Similar to NutNet standard but top strand at 1.2 m.	Sheep, wild herbivores
spin.us	Similar to NutNet standard with two modifications: 3.6 cm hardware cloth and 1.5 m high fences.	Wild herbivores
valm.ch	2.7 m wooden poles (25 cm diameter) driven 70 cm into ground, 3 m apart, covered with 5 cm square mesh to 2 m high and with extra cabling and supports to prevent snow damage. Fences enclose 6 m x 7 m area.	Wild herbivores

Supplementary Table 3A. Model testing the effects of nutrient addition and fencing and their interactions on aboveground community biomass in n=58 sites with two to four years of response. The main effect values represent model mean differences of the experimental treatment from the control after controlling for site and year as random effects. The nutrient by fence interaction ("Nut*Fnc") is compared to the sum of the effect of nutrients alone and fencing alone. Linear mixed-effects model was fit by maximum likelihood. Random effects in the model were 'site' (s.d.= 0.31) and 'treatment year nested within site' (s.d.=0.19). The intercept is the mean value of the unfenced and unfertilized control plots. Live biomass was $\log_{10}$ transformed. Table shows parameter estimate, standard error, degrees of freedom, test statistic, and p-value for the 2-tailed test.
Model formula: lm.lg ~ fert * exclose + (1 | site/year_trt)

	Estimate	Std. Error	DF	t-value	p-value
(Intercept)	2.39	4.35×10^{-2}	60	54.86	< 0.001
Fertilization (Nut)	0.14	1.21×10^{-2}	2513	11.61	< 0.001
Fencing (Fnc)	4.18×10^{-2}	1.23×10^{-2}	2515	3.40	< 0.001
Nut x Fnc	2.26×10^{-2}	1.74×10^{-2}	2513	1.30	0.194

Supplementary Table 3B. Model testing the effects of nutrient addition and fencing and their interactions on aboveground community biomass in n=42 sites with five to seven years of response. The main effect values represent model mean differences of the experimental treatment from the control after controlling for site and year as random effects. The nutrient by fence interaction ("Nut*Fnc") is compared to the sum of the effect of nutrients alone and fencing alone. Linear mixed-effects model was fit by maximum likelihood. Random effects in the model were 'site' (s.d.= 0.33) and 'treatment year nested within site' (s.d.=0.25). The intercept is the mean value of the unfenced and unfertilized control plots. Live biomass was log_{10} transformed. Table shows parameter estimate, standard error, degrees of freedom, test statistic, and p-value for the 2-tailed test.
Model formula: lm.lg ~ fert * exclose + (1 | site/year_trt)

	**Estimate**	**Std. Error**	**DF**	**t-value**	**p-value**
(Intercept)	2.34	0.05	41.9	43.42	< 0.001
Fertilization (Nut)	0.17	0.01	3074	14.14	< 0.001
Fencing (Fnc)	0.05	0.01	3074	4.58	< 0.001
Nut x Fnc	-0.02	0.02	3073	-1.00	0.320

Supplementary Table 3C. Model testing the effects of nutrient addition and fencing and their interactions on aboveground community biomass in n=24 sites with eight to ten years of response. The main effect values represent model mean differences of the experimental treatment from the control after controlling for site and year as random effects. The nutrient by fence interaction ("Nut*Fnc") is compared to the sum of the effect of nutrients alone and fencing alone. Linear mixed-effects model was fit by maximum likelihood. Random effects in the model were 'site' (s.d.= 0.24) and 'treatment year nested within site' (s.d.=0.27). The intercept is the mean value of the unfenced and unfertilized control plots. Live biomass was log_{10} transformed. Table shows parameter estimate, standard error, degrees of freedom, test statistic, and p-value for the 2-tailed test.
Model formula: lm.lg ~ fert * exclose + (1 | site/year_trt)

	**Estimate**	**Std. Error**	**DF**	**t-value**	**p-value**
(Intercept)	2.33	0.05	23.6	43.69	< 0.001
Fertilization (Nut)	0.20	0.01	2750	16.82	< 0.001
Fencing (Fnc)	0.06	0.01	2751	4.87	< 0.001
Nut x Fnc	-0.03	0.02	2750	-1.99	0.047

Supplementary Table 3D. Model testing the effects of nutrient addition and fencing and their interactions on aboveground community biomass in n=8 sites with domestic grazing with two to four years of response. The main effect values represent model mean differences of the experimental treatment from the control after controlling for site and year as random effects. The nutrient by fence interaction ("Nut*Fnc") is compared to the sum of the effect of nutrients alone and fencing alone. Linear mixed-effects model was fit by maximum likelihood. Random effects in the model were 'site' (s.d.= 0.41) and 'treatment year nested within site' (s.d.=0.17). The intercept is the mean value of the unfenced and unfertilized control plots. Live biomass was log_{10} transformed. Table shows parameter estimate, standard error, degrees of freedom, test statistic, and p-value for the 2-tailed test.
Model formula: lm.lg ~ fert * exclose + (1 | site/year_trt)

	**Estimate**	**Std. Error**	**DF**	**t-value**	**p-value**
(Intercept)	2.13	0.15	7.3	14.27	< 0.001
Fertilization (Nut)	0.09	0.04	323.1	2.37	0.019
Fencing (Fnc)	0.07	0.03	323.2	1.75	0.081
Nut x Fnc	0.15	0.05	323.1	2.79	0.006

Supplementary Table 4A. Effects of site biotic factors – an empirical herbivore index, modeled grazer biomass, site-level plant species richness, and species turnover – on plant biomass in response to experimental fencing and fertilization treatments after two treatment years (to maximize spatial extent). Models included the subset of n=51 sites with values for all model covariates. Linear mixed-effects model was fit by maximum likelihood. Random effects in the model were 'site' (s.d.= 0.28) and 'treatment year nested within site' (s.d.=0.19). The intercept is the mean value of the unfenced and unfertilized control plots. Live biomass was log_{10} transformed and the grazing intensity index was square root transformed. Table shows parameter estimate, standard error, degrees of freedom, test statistic, and p-value for the 2-tailed test. Model formula: lm.lg ~ (fert * exclose) * (site_richness + meanJacc + grazer.biomass + herb.index.sq) + (1 | site/year_trt)

	Estimate	Std. Error	DF	t-value	p-value
(Intercept)	2.97	0.20	56.43	15.12	< 0.001
Fertilization (Nut)	0.11	0.07	2261	1.55	0.121
Fencing (Fnc)	-0.02	0.07	2263	-0.30	0.763
Site richness (Rich)	-3.01×10^{-3}	1.53×10^{-3}	55.7	-1.97	0.053
Species turnover (Jacc)	0.14	0.07	2295	1.98	0.048
Modeled grazer biomass (MGraz)	7.41×10^{-3}	0.04	55.43	0.17	0.864
Herbivore index (Intens)	-0.18	0.05	54.17	-3.56	0.001
Nut x Fnc	-0.06	0.10	2261	-0.63	0.528
Nut x Rich	5.64×10^{-4}	4.85×10^{-4}	2261	1.16	0.245
Nut x Jacc	0.09	0.09	2263	1.06	0.291
Nut x MGraz	0.02	0.01	2262	1.67	0.096
Nut x Intens	-0.03	0.02	2261	-1.91	0.057
Fnc x Rich	1.20×10^{-4}	4.94×10^{-4}	2262	0.24	0.808
Fnc x Jacc	-0.07	0.09	2264	-0.78	0.436
Fnc x MGraz	4.38×10^{-3}	0.01	2262	0.33	0.745
Fnc x Intens	0.03	0.02	2262	1.87	0.062
Nut x Fnc x Rich	2.45×10^{-4}	6.97×10^{-4}	2261	0.35	0.725
Nut x Fnc x Jacc	-0.09	0.13	2264	-0.71	0.476
Nut x Fnc x MGraz	-0.03	0.02	2262	-1.33	0.184
Nut x Fnc x Intens	0.06	0.02	2261	2.52	0.012

Supplementary Table 4B. Effects of climate (mean annual precipitation, mean annual temperature, mean precipitation in the warmest quarter), an empirical herbivore index, and modeled grazer biomass on biomass in response to experimental fencing and fertilization treatments after two treatment years (to maximize spatial extent). Models included covariates available for the subset of n=51 sites with values for all model covariates. Linear mixed-effects model was fit by maximum likelihood. Random effects in the model were 'site' (s.d.= 0.25) and 'treatment year nested within site' (s.d.=0.19). The intercept is the mean value of the unfenced and unfertilized control plots. Live biomass was log_{10} transformed. Table shows parameter estimate, standard error, degrees of freedom, test statistic, and p-value for the 2-tailed test. Model formula: lm.lg ~ (fert * exclose) * (map + mat + map_warm_q + site_richness + grazer.biomass + herb.index.sq) + (1 | site/year_trt)

	Estimate	Std. Error	DF	t-value	p-value
(Intercept)	2.56	0.25	55.82	10.42	< 0.001
Fertilization (Nut)	0.08	0.09	2264	0.89	0.374
Fencing (Fnc)	0.03	0.09	2264	0.30	0.765
Mean annual precipitation (MAP)	1.41×10^{-4}	1.22×10^{-4}	55.47	1.16	0.252
Mean annual temperature (MAT)	5.68×10^{-3}	7.04×10^{-3}	55.62	0.81	0.423
MAP warmest quarter (MAPWQ)	6.58×10^{-4}	3.36×10^{-4}	55.65	1.96	0.055
Site richness (Rich)	-1.90×10^{-3}	1.42×10^{-3}	55.49	-1.33	0.188
Modeled grazer biomass (MGraz)	-3.23×10^{-3}	0.04	55.68	-0.08	0.935
Grazing intensity index (Intens)	-0.15	0.05	55.05	-2.99	0.004
Nut x Fnc	0.07	0.12	2263	0.54	0.591
Nut x MAP	1.63×10^{-5}	4.12×10^{-5}	2264	0.40	0.692
Nut x MAT	4.42×10^{-3}	2.37×10^{-3}	2264	1.87	0.062

Nut x MAPWQ	-5.56 x10^{-5}	1.12 x10^{-4}	2263	-0.50	0.620
Nut x Rich	7.04 x10^{-4}	4.70 x10^{-4}	2263	1.50	0.134
Nut x MGraz	0.02	0.01	2264	1.76	0.078
Nut x Intens	-0.02	0.02	2263	-1.49	0.137
Fnc x MAP	-1.22 x10^{-5}	4.16 x10^{-5}	2264	-0.29	0.769
Fnc x MAT	-3.50 x10^{-3}	2.38 x10^{-3}	2265	-1.48	0.140
Fnc x MAPWQ	-2.50 x10^{-5}	1.13 x10^{-4}	2266	-0.22	0.826
Fnc x Rich	-1.44 x10^{-4}	4.75 x10^{-4}	2264	-0.30	0.761
Fnc x MGraz	9.69 x10^{-3}	0.01	2264	0.73	0.464
Fnc x Intens	0.03	0.02	2264	1.49	0.137
Nut x Fnc x MAP	-1.44 x10^{-4}	5.86 x10^{-5}	2264	-2.45	0.014
Nut x Fnc x MAT	-9.56 x10^{-4}	3.37 x10^{-3}	2265	-0.28	0.777
Nut x Fnc x MAPWQ	1.34 x10^{-4}	1.60 x10^{-4}	2263	0.84	0.403
Nut x Fnc x Rich	-1.02 x10^{-4}	6.71 x10^{-4}	2264	-0.15	0.880
Nut x Fnc x MGraz	-0.03	0.02	2264	-1.48	0.139
Nut x Fnc x Intens	0.04	0.02	2263	1.72	0.086

Supplementary Table 4C. Effects of plot-scale soil chemistry, site-scale nitrogen deposition rate, an empirical herbivore index, and modeled grazer biomass on biomass in response to experimental fencing and fertilization treatments after two treatment years (to maximize spatial extent). Models included covariates available for a subset of n=36 sites. Linear mixed-effects model was fit by maximum likelihood. Random effects in the model were 'site' (s.d.= 0.20) and 'treatment year nested within site' (s.d.=0.20). The intercept is the mean value of the unfenced and unfertilized control plots. Live biomass was log_{10} transformed. Table shows parameter estimate, standard error, degrees of freedom, test statistic, and p-value for the 2-tailed test. Model formula: lm.lg ~ (fert * exclose) * (ndep + ppm_n + ppm_p + ph + herb.index.sq + grazer.biomass + site_richness) + (1 | site/year_trt)

	Estimate	Std. Error	DF	t-value	p-value
(Intercept)	2.70	0.24	80.7	11.01	< 0.001
Fertilization (Nut)	0.06	0.17	1505	0.37	0.714
Fencing (Fnc)	-0.22	0.17	1507	-1.36	0.176
N deposition (Ndep)	0.02	6.13 x10^{-3}	43.35	3.74	0.001
Nitrogen (N)	4.73 x10^{-6}	6.13 x10^{-6}	1004	0.77	0.441
Phosphorus (P)	3.82 x10^{-4}	5.23 x10^{-4}	957.9	0.73	0.466
pH (pH)	-0.04	0.02	1315	-1.63	0.103
Grazing intensity index (Intens)	-0.11	0.05	39.09	-2.09	0.043
Modeled grazer biomass (MGraz)	0.07	0.04	39.97	1.47	0.149
Site richness (Rich)	-2.09 x10^{-3}	1.52 x10^{-3}	39.13	-1.37	0.178
Nut x Fnc	0.02	0.23	1508	0.09	0.930
Nut x Ndep	1.70 x10^{-3}	2.86 x10^{-3}	1512	0.59	0.552
Nut x N	-2.13 x10^{-6}	5.94 x10^{-6}	1514	-0.36	0.720
Nut x P	-1.73 x10^{-4}	4.30 x10^{-4}	1507	-0.40	0.688
Nut x pH	0.03	0.02	1506	1.31	0.190
Nut x Intens	-0.06	0.02	1504	-2.72	0.007
Nut x MGraz	0.03	0.02	1506	1.55	0.122
Nut x Rich	5.70 x10^{-4}	6.23 x10^{-4}	1504	0.91	0.361
Fnc x Ndep	-2.37 x10^{-3}	2.98 x10^{-3}	1511	-0.80	0.426
Fnc x N	1.64 x10^{-5}	5.93 x10^{-6}	1507	2.77	0.006
Fnc x P	2.25 x10^{-5}	4.32 x10^{-4}	1505	0.05	0.958
Fnc x pH	7.19 x10^{-3}	0.02	1508	0.31	0.756
Fnc x Intens	0.04	0.02	1507	1.60	0.111
Fnc x MGraz	0.03	0.02	1511	1.44	0.149
Fnc x Rich	7.41 x10^{-4}	6.35 x10^{-4}	1506	1.17	0.243
Nut x Fnc x Ndep	-1.76 x10^{-3}	4.11 x10^{-3}	1507	-0.43	0.669
Nut x Fnc x N	-1.50 x10^{-5}	8.58 x10^{-6}	1509	-1.75	0.080
Nut x Fnc x P	-2.00 x10^{-4}	6.15 x10^{-4}	1504	-0.33	0.745
Nut x Fnc x pH	-0.01	0.03	1509	-0.34	0.733
Nut x Fnc x Intens	0.07	0.03	1505	2.12	0.034
Nut x Fnc x MGraz	-0.03	0.03	1509	-1.04	0.301
Nut x Fnc x Rich	-1.93 x10^{-4}	8.89 x10^{-4}	1505	-0.22	0.828

Supplementary Figure 1. Among-year dynamics show interannual variability in mean grassland biomass in control plots and in response to fertilization and fencing treatments. Error bars show 1 standard error.

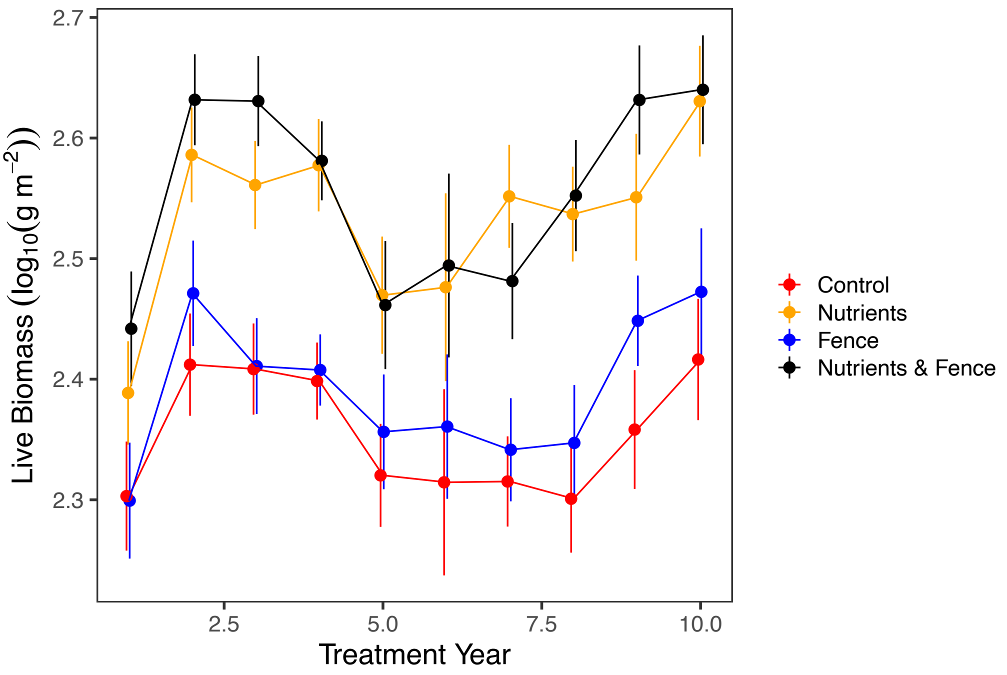

Supplementary Figure 2. Site-level effects of herbivore removal on grassland biomass with and without elevated nutrient supply. Numbers identify sites (SupplementaryTable 1), and the 1:1 relationship is shown as a solid black line. These results are broadly consistent regardless of the number of experimental years, so we show results across the greatest spatial extent (n=58 sites), representing 2-3 years of treatment responses.

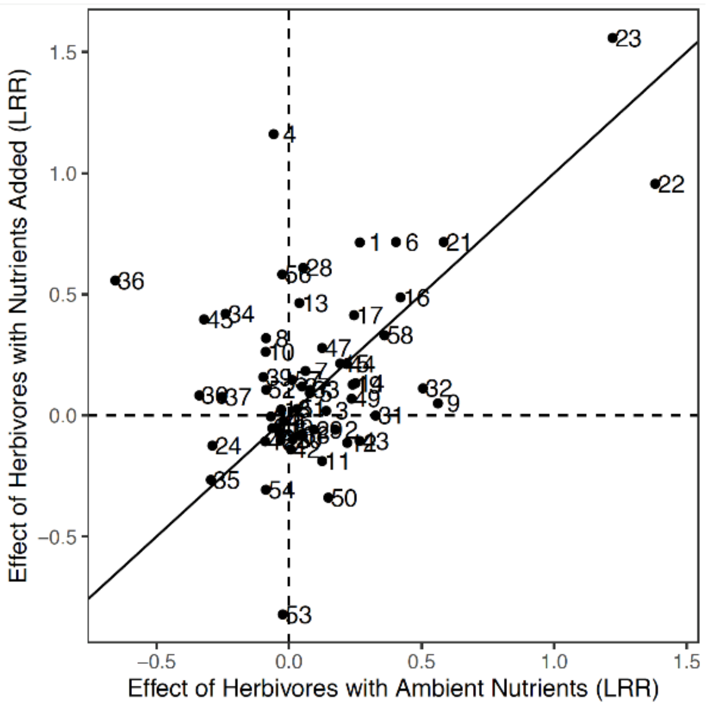

Supplementary Figure 3. Treatment effects on aboveground grassland biomass after two to four years of response at the n=8 sites with both domestic livestock and wild herbivores (full model shown in Supplementary Table 3D). Error bars represent 95% confidence intervals. Main effect values represent model mean differences of the experimental treatment from the control after controlling for site and year as random effects. The nutrient by fence interaction ("Nut*Fnc") is compared to the sum of the effect of nutrients alone and fencing alone, thus the substantial "Nut*Fnc" term indicates that herbivores consume more biomass under fertilized than ambient conditions.

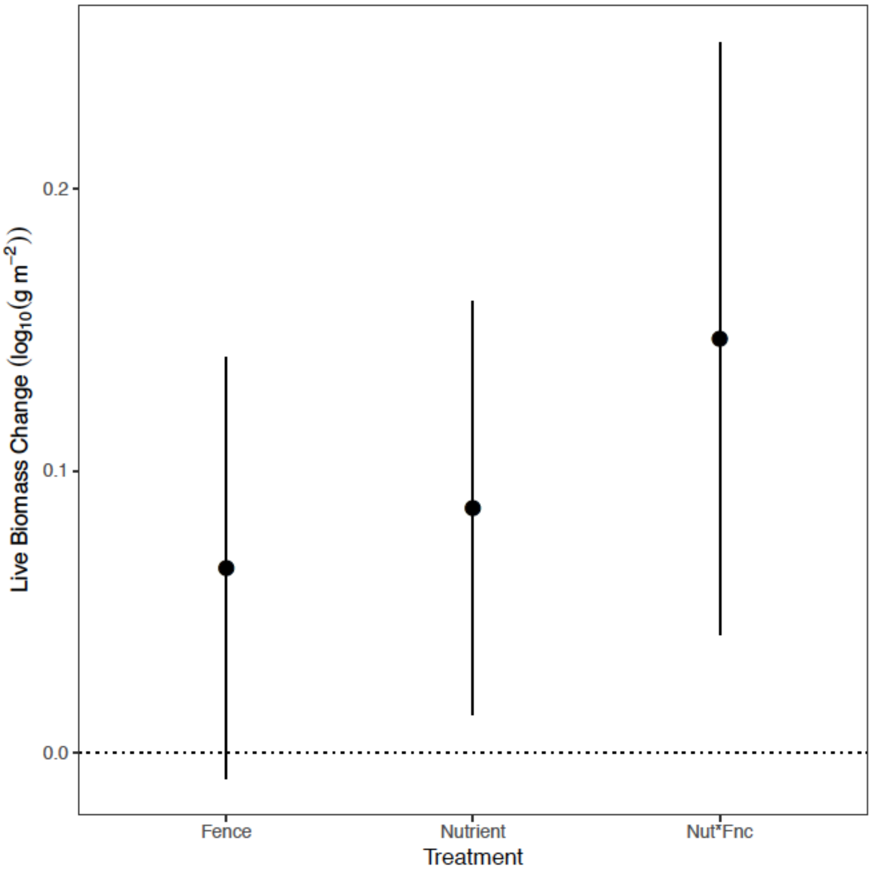

Supplementary Table 5. Author contributions and site-level acknowledgments. Site names match those in Supplementary Table 1.

Name	Site(s) used in analysis	Developed and framed research question(s)	Analyzed data	Contributed to data analyses	Wrote the paper	Contributed to paper writing	Site coordinator	Nutrient Network coordinator
Borer, Elizabeth	bnch.us, cdcr.us, hopl.us, look.us, mcla.us, sier.us	x	x		x		x	x
Harpole, W. Stanley	cbgb.us, sier.us			x		x	x	
Adler, Peter B.	shps.us					x	x	
Bugalho, Miguel	comp.pt					x	x	
Cadotte, Marc W.	koffler.ca					x	x	
Caldeira, Maria Conceição	comp.pt					x	x	
Campana, Sofía	chilcas.ar					x		
Arnillas, Carlos Alberto	koffler.ca					x	x	
Dickman, Chris R.						x	x	
Dickson, Timothy L.	glcr.us					x	x	
Donohue, Ian	burren.ie					x	x	
Eskelinen, Anu	kilp.fi, saana.fi					x	x	
Firn, Jennifer	burrawan.au					x	x	
Graff, Pamela	chilcas.ar					x		
Gruner, Daniel	sage.us					x	x	
Heckman, Robert W.	unc.us					x	x	
Koltz, Amanda	tyso.us					x		
Komatsu, Kimberly	konz.us, saline.us					x	x	
Lannes, Luciola	lagoas.br					x	x	
MacDougall, Andrew S.	cowi.ca					x	x	
Martina, Jason P.						x	x	
Moore, Joslin L.	bogong.au					x	x	
Mortensen, Brent	cbgb.us					x	x	
Ochoa-Hueso, Raúl	yarra.au					x		
Olde Venterink, Harry	lagoas.br					x	x	
Power, Sally	yarra.au					x	x	
Price, Jodi	ping.au					x	x	
Risch, Anita C.	valm.ch					x	x	
Sankaran, Mahesh	kibber.in					x	x	
Schütz, Martin	valm.ch					x	x	
Sitters, Judith						x		
Stevens , Carly	lancs.uk					x	x	
Virtanen, Risto	kilp.fi, saana.fi					x	x	
Wilfahrt, Peter A.	bayr.de					x	x	
Seabloom, Eric	bnch.us, cdcr.us, hopl.us, look.us, mcla.us, sier.us	x	x			x	x	x

Supplementary Table 6. Principal investigators of sites contributing data but who are not authors, listed by site; site names match those in Supplementary Table 1. Their effort in providing samples was critical to this work.

Site PI	Site code
Elizabeth Boughton	arch.us
Guozhen Du	azi.cn
David Wedin	barta.us
Anke Jentsch, Marie Spohn	bayr.de
Kendi Davies, Brett Melbourne	bldr.us
Johannes Knops	cdpt.us
Amandine Hansart, Beatriz Decencière	cereep.fr
Laura Yahdjian, Enrique Chaneton	chilcas.ar
Elsa Cleland	elliot.us
Sabine Güsewell	frue.ch
Peter Wragg	gilb.za
Rebecca McCulley	hall.us,
Nicole DeCrappeo , David Pyke	hart.us
Michael Crawley	hero.uk,
Elizabeth Wolkovich, Kathryn Cottingham	hnvr.us
Lars Brudvig	kbs.us
John Morgan	kiny.au
Pedro Daleo, Juan Alberti	marc.ar
Suzanne Prober	mtca.au
Helmut Hillebrand	pape.de
John Dwyer	pinj.au
Pablo Peri, Hector Bahamonde	potrok.ar
Ellen Damschen, John Orrock	sava.us
T. Michael Anderson	sereng.tz
Cynthia Brown, Julia Klein, Dana Blumenthal	sgs.us
Janneke Hille Ris Lambers, Jonathan Bakker	smith.us
Andrew Leakey	trel.us
Maria Silveira	ufrec.us
